# Targeting Inflammation After Hemorrhagic Shock as a Molecular and Experimental Journey to Improve Outcomes: A Review

**DOI:** 10.7759/cureus.77776

**Published:** 2025-01-21

**Authors:** Kenneth Meza Monge, Astrid Ardon-Lopez, Akshay Pratap, Juan-Pablo Idrovo

**Affiliations:** 1 Department of Surgery, Division of GI, Trauma, and Endocrine Surgery, University of Colorado, Aurora, USA; 2 Department of Surgery, Division of Plastic and Reconstructive Surgery, University of Colorado, Aurora, USA

**Keywords:** endothelial dysfunction, hemorrhagic shock, inflammation, molecular pathways, therapeutic strategies

## Abstract

Hemorrhagic shock continues to be a major contributor to trauma-related fatalities globally, posing a significant and intricate pathophysiological challenge. The condition is marked by injury and blood loss, which activate molecular cascades that can quickly become harmful. The inflammatory response exhibits a biphasic pattern, beginning with a hyper-inflammatory phase that transitions into immunosuppression, posing significant obstacles to effective therapeutic interventions. This review article explores the intricate molecular mechanisms driving inflammation in hemorrhagic shock, emphasizing cellular signaling pathways, endothelial dysfunction, and immune activation. We discuss the role of molecular biomarkers in tracking disease progression and stratifying risk, with a focus on markers of endothelial dysfunction and inflammatory mediators as potential prognostic tools.

Additionally, we assess therapeutic strategies, spanning traditional approaches like hemostatic resuscitation to advanced immunomodulatory treatments. Despite promising advancements in molecular monitoring and targeted therapies, challenges persist in bridging experimental findings with clinical applications. Future efforts must prioritize understanding the dynamic progression of inflammatory pathways and refining the timing of interventions to improve outcomes in hemorrhagic shock management.

## Introduction and background

The molecular pathophysiology of hemorrhagic shock represents one of the most complex challenges in critical care medicine, with its devastating impact reflected in mortality rates of 30-40% among trauma patients worldwide and approximately 60,000 deaths annually in the United States alone [1]. Understanding the intricate molecular mechanisms driving inflammation after hemorrhagic shock is imperative for several critical reasons. First, the initial tissue injury and hypoperfusion trigger a cascade of molecular events that extend far beyond simple blood loss, encompassing complex interactions between the endothelium, immune system, and coagulation pathways that ultimately determine patient survival [2].

The importance of exploring molecular mechanisms behind post-hemorrhagic shock inflammation stems from the observation that the inflammatory response often proves more lethal than the original injury. This response involves sophisticated molecular crosstalk between damage-associated molecular patterns (DAMPs), pattern recognition receptors, and inflammatory mediators that orchestrate both protective and destructive processes [3]. The temporal evolution of these molecular pathways, transitioning from an initial hyper-inflammatory state to a potentially devastating immunosuppressive phase, creates unique challenges for therapeutic intervention and necessitates precise molecular monitoring.

Identifying and utilizing biomarkers to monitor inflammation after hemorrhagic shock has become crucial for several reasons. These molecular indicators provide real-time information about disease progression, help predict outcomes, and guide therapeutic interventions [4]. Developing biomarkers that accurately reflect endothelial dysfunction, immune activation, and organ damage has opened new possibilities for personalized treatment approaches [5]. Furthermore, these molecular markers offer crucial insights into the complex pathophysiology of hemorrhagic shock, helping bridge the gap between experimental findings and clinical applications.

The development of therapies targeting specific molecular aspects of inflammation in hemorrhagic shock represents a critical frontier in improving patient outcomes. Traditional approaches focusing solely on volume replacement and hemodynamic stabilization fail to address the underlying molecular cascades that often determine patient survival [6]. Novel therapeutic strategies targeting specific inflammatory mediators, signaling pathways, and cellular responses offer the potential for more precise intervention. However, the successful development of these targeted therapies requires a thorough understanding of the molecular mechanisms involved in both the initiation and resolution of inflammation [7].

This comprehensive review examines the intricate molecular pathways involved in post-hemorrhagic shock inflammation, focusing specifically on experimental findings illuminating potential therapeutic targets. We begin by exploring the complex cascade of inflammatory mediators and cellular responses that characterize the condition, with particular attention to molecular mechanisms identified in experimental models. The review then analyzes current and emerging molecular biomarkers for patient monitoring and risk stratification and then evaluates therapeutic strategies targeting specific aspects of the inflammatory response. Throughout, we emphasize the translation of molecular insights from experimental studies to potential clinical applications, highlighting both promising advances and critical challenges in the field.

Critical to our discussion is the recognition that current experimental models have revealed novel molecular pathways altered after hemorrhagic shock, which modulate inflammation and affect outcomes. These findings provide crucial insights into potential therapeutic targets while highlighting the challenges of translating molecular interventions from controlled experimental settings to the complex clinical environment. This review aims to provide a foundation for developing more effective, targeted approaches to managing inflammation in hemorrhagic shock by focusing on molecular mechanisms and experimental evidence.

## Review

Methodology

A thorough literature search was conducted using Medline, PubMed, Google Scholar, and Science Direct to identify relevant publications from January 1980 to December 2024 addressing inflammation in hemorrhagic shock. The search included studies without restrictions on language, population demographics, or publication type, encompassing articles, books, and book chapters to ensure a comprehensive review. The focus was on molecular mechanisms of inflammation, biomarkers for disease progression, and therapeutic strategies targeting inflammatory pathways. Key search terms included “hemorrhagic shock”, “inflammation”, “endothelial dysfunction”, “molecular pathways”, “biomarkers”, and “therapeutic strategies”. Inclusion criteria were broad, incorporating studies on various populations, experimental and clinical research, systematic reviews, meta-analyses, and relevant academic texts. Exclusion criteria were limited to studies unrelated to hemorrhagic shock or inflammation. Two independent reviewers critically assessed the selected materials based on methodological quality, consistency of findings, and relevance to the study objectives. Discrepancies were resolved through consensus discussions, ensuring a rigorous and inclusive review process. The findings from this review serve as the foundation for the analysis presented in this study.

Molecular mechanisms of the inflammatory response in hemorrhagic shock

Early Events in the Inflammatory Cascade

The pathophysiology of hemorrhagic shock represents an intricate interplay between vascular injury, immune activation, and organ dysfunction [8]. Research demonstrates that acute blood loss exceeding 20% of total blood volume (approximately 1000 mL in adults) initiates early inflammatory responses, while losses greater than 30-40% trigger profound systemic inflammation [9]. These thresholds, identified through experimental models and clinical studies, correspond to Class III and IV hemorrhage in the traditional classification system and consistently activate inflammatory cascades even when blood pressure remains relatively preserved through compensatory mechanisms [10].

The inflammatory cascade begins with mechanical trauma to blood vessels, causing direct damage to endothelial cells and resident immune cells within the vessel wall [11]. Injured endothelial cells rapidly release pre-stored inflammatory mediators, including platelet-selectin (P-selectin) and von Willebrand factor (vWF), from specialized storage organelles called Weibel-Palade bodies [12]. Simultaneously, these activated endothelial cells synthesize and secrete monocyte chemoattractant protein-1 (MCP-1/CCL2). Research using experimental models demonstrates that this endothelial activation occurs within minutes of injury, establishing initial chemotactic gradients that guide immune cell recruitment to damaged tissues [13].

Tissue-resident macrophages and mast cells, sensing mechanical damage through specialized mechanoreceptors, initiate a secondary wave of inflammatory mediator production. These cells release tumor necrosis factor-alpha (TNF-α), interleukin-1 alpha (IL-1α), histamine, and the neutrophil-attracting chemokines C-X-C motif ligand 1 and 2 (CXCL1/CXCL2) [14]. The combination of these mediators creates a sophisticated molecular network that amplifies the initial inflammatory response while recruiting additional immune cells to the site of injury.

Endothelial Glycocalyx Degradation: A Critical Molecular Event

Endothelial glycocalyx degradation emerges as a critical molecular event in early hemorrhagic shock [15]. Pro-inflammatory cytokines, particularly TNF-α, interleukin-1 beta (IL-1β), and interleukin-6 (IL-6) activate matrix metalloproteinases-2 and -9 (MMP-2, MMP-9) and heparanase [16]. These enzymes systematically degrade the protective glycocalyx layer, exposing the underlying subendothelial matrix. This exposure reveals tissue factors and collagen, creating a molecular bridge between inflammation and coagulation activation. Experimental studies demonstrate that the extent of glycocalyx degradation correlates directly with organ dysfunction severity and mortality rates [15].

Platelet Activation and Molecular Interactions

The exposed subendothelial matrix initiates platelet activation through specific receptor-ligand interactions. Platelet glycoprotein VI (GPVI) and integrin α2β1 bind directly to exposed collagen, while the glycoprotein Ib-V-IX (GPIb-V-IX) complex interacts with vWF [17]. These molecular interactions trigger platelet degranulation, releasing densely packed granules containing serotonin, adenosine diphosphate (ADP), and adenosine triphosphate (ATP). Additionally, platelets release α-granules rich in platelet-derived growth factor (PDGF), platelet factor 4 (PF4/CXCL4), and regulated upon activation, normal T cell expressed and secreted (RANTES/CCL5) factor [18].

Cell Death Pathways and the Release of DAMPs

Cellular injury during hemorrhagic shock induces regulated cell death through distinct molecular pathways. Receptor-interacting serine/threonine-protein kinase 1 and 3 (RIPK1/RIPK3) and mixed lineage kinase domain-like pseudokinase (MLKL) mediate necroptosis, while caspase-1 and gasdermin D orchestrate pyroptosis [19]. These death pathways release specific damage-associated molecular patterns (DAMPs), including high mobility group box 1 (HMGB1), histones H3 and H4, and mitochondrial components. These DAMPs interact with pattern recognition receptors, particularly toll-like receptor 4 (TLR4) and receptor for advanced glycation end products (RAGE), initiating distinct but overlapping inflammatory signaling cascades [20].

Mitochondrial Dysfunction and Oxidative Stress

Tissue hypoperfusion from blood loss exceeding 30% of total volume forces a metabolic shift from aerobic to anaerobic pathways, creating a distinct molecular environment that shapes inflammatory responses [21]. Mitochondrial dysfunction plays a central role as electron transport chain efficiency diminishes, particularly at complexes I and III. The resulting electron leakage generates superoxide radicals, subsequently converted to hydrogen peroxide by mitochondrial superoxide dismutase [22]. This oxidative stress compounds with ATP depletion to disrupt cellular ion homeostasis, particularly affecting calcium handling through compromised ATP-dependent ion pumps. The resulting calcium accumulation further damages mitochondria, establishing a destructive cycle of organelle dysfunction and reactive oxygen species (ROS) production [23].

Cytosolic sources complement mitochondrial ROS production through specific enzymatic pathways. Nicotinamide adenine dinucleotide phosphate oxidases (NOX), particularly NOX2 in neutrophils and NOX4 in endothelial cells, generate substantial amounts of superoxide and hydrogen peroxide [24]. Xanthine oxidase, converted from xanthine dehydrogenase under hypoxic conditions, produces additional ROS while metabolizing purines from degraded ATP. The uncoupling of endothelial nitric oxide synthase (eNOS), driven by tetrahydrobiopterin depletion, leads to superoxide production instead of the usual nitric oxide synthesis [25].

Coagulation Dynamics and Trauma-Induced Coagulopathy

The coagulation response in hemorrhagic shock exhibits distinct phases that evolve as blood loss progresses. Initial tissue factor exposure triggers the extrinsic coagulation pathway through tissue factor-factor VIIa complex formation [26]. Thrombin generation predominates early, catalyzing fibrinogen conversion to fibrin while simultaneously activating platelets through protease-activated receptors (PARs), particularly PAR-1 and PAR-4. This activation creates a prothrombotic surface, supporting further platelet adhesion and thrombus formation [27]. As hemorrhagic shock progresses beyond 40% blood volume loss, coagulation often becomes dysregulated through protein C activation and hyperfibrinolysis, leading to trauma-induced coagulopathy [28].

Complement System Activation in Severe Hemorrhage

The complement system intersects with coagulation at multiple points during severe hemorrhage. Thrombin directly cleaves complement components C3 and C5, generating anaphylatoxins C3a and C5a independent of traditional complement activation pathways [29]. These complement fragments enhance vascular permeability and promote leukocyte chemotaxis while amplifying platelet activation. P-selectin expression on activated platelets triggers additional complement activation, creating a positive feedback loop between coagulation and complement systems [30].

Microvascular Thrombosis and Neutrophil Extracellular Traps

Microvascular thrombosis develops through neutrophil extracellular trap (NET) formation, a process requiring peptidyl arginine deiminase 4 (PAD4)-mediated chromatin decondensation. These NETs provide a scaffold for platelet adhesion and fibrin deposition while activating the contact pathway of coagulation through their negatively charged DNA components [31]. The resulting microthrombi, composed of platelets, fibrin, and entrapped leukocytes, obstruct capillary blood flow, exacerbating tissue hypoxia and promoting inflammation through ischemia-reperfusion injury.

Neurological Implications of Systemic Inflammation

The central nervous system exhibits distinct vulnerability to systemic inflammation during hemorrhagic shock through coordinated molecular mechanisms. Blood-brain barrier disruption occurs as circulating TNF-α and IL-1β activate brain endothelial cells and weaken tight junctions, allowing peripheral inflammatory molecules and immune cells to infiltrate the brain parenchyma [32]. This triggers microglial and astrocyte activation, characterized by morphological changes and increased production of pro-inflammatory mediators. These activated cells release additional TNF-α, IL-1β, and nitric oxide while astrocytes increase the expression of glial fibrillary acidic protein, creating a self-sustaining cycle of neuroinflammation [33].

Compensatory Anti-Inflammatory Response Syndrome (CARS)

The inflammatory cascade in hemorrhagic shock eventually triggers compensatory anti-inflammatory mechanisms that attempt to restore homeostasis but can lead to dangerous immunosuppression [34]. This compensatory anti-inflammatory response syndrome (CARS) represents the complex reprogramming of the immune system involving multiple cell types and molecular mediators [35]. Experimental studies demonstrate that this transition typically occurs 24-48 hours after initial blood loss, though timing varies based on injury severity and resuscitation adequacy [36].

Immune Cell Reprogramming During CARS

Monocytes undergo significant phenotypic changes during CARS, characterized by decreased human leukocyte antigen-DR (HLA-DR) expression and reduced antigen presentation capacity [37]. This reprogramming involves epigenetic modifications affecting promoter regions of pro-inflammatory genes through increased DNA methylation and decreased histone acetylation [38]. These cells simultaneously increase the production of anti-inflammatory mediators, notably interleukin-10 (IL-10) and transforming growth factor-beta (TGF-β), while reducing their responsiveness to inflammatory stimuli through down-regulation of pattern recognition receptors [37].

T lymphocyte populations undergo substantial alterations during CARS. The T cell repertoire shifts toward a predominance of regulatory T cells (Tregs) expressing the transcription factor forkhead box P3 (FoxP3) [39-41]. These expanded Tregs actively suppress other immune cells through both contact-dependent mechanisms and IL-10/TGF-β secretion [42,43]. Conventional T cells exhibit increased apoptosis rates, particularly affecting CD4+ and CD8+ effector populations, leading to significant lymphopenia [44,45]. Surviving T cells show impaired proliferation responses to antigens and decreased production of pro-inflammatory cytokines, especially interferon-gamma (IFN- γ) and interleukin-2 (IL-2) [46].

Role of Myeloid-Derived Suppressor Cells (MDSCs)

Myeloid-derived suppressor cells (MDSCs) emerge as critical mediators of immunosuppression during CARS. These immature myeloid cells expand in circulation and accumulate in tissues, suppressing both innate and adaptive immune responses through multiple mechanisms [47]. MDSCs produce arginase-1 (ARG-1), and depleting arginine is necessary for T-cell function. It also generates ROS that inhibits T-cell receptor signaling [47-49]. Additionally, they produce indoleamine 2,3-dioxygenase, which catabolizes tryptophan and generates immunosuppressive metabolites that further dampen immune responses [50,51].

Endothelial Phenotypic Shifts During CARS

The endothelial response shifts during CARS, characterized by decreased adhesion molecule expression and reduced responsiveness to inflammatory signals [52]. This change occurs through the induction of negative regulators of inflammation, including A20 and suppressor of cytokine signaling (SOCS) proteins, which dampen nuclear factor kappa B (NF-κB) signaling and cytokine receptor activation [53,54]. The altered endothelial phenotype reduces leukocyte trafficking and decreases pro-inflammatory mediator production, contributing to the overall immunosuppressive state.

**Figure 1:**
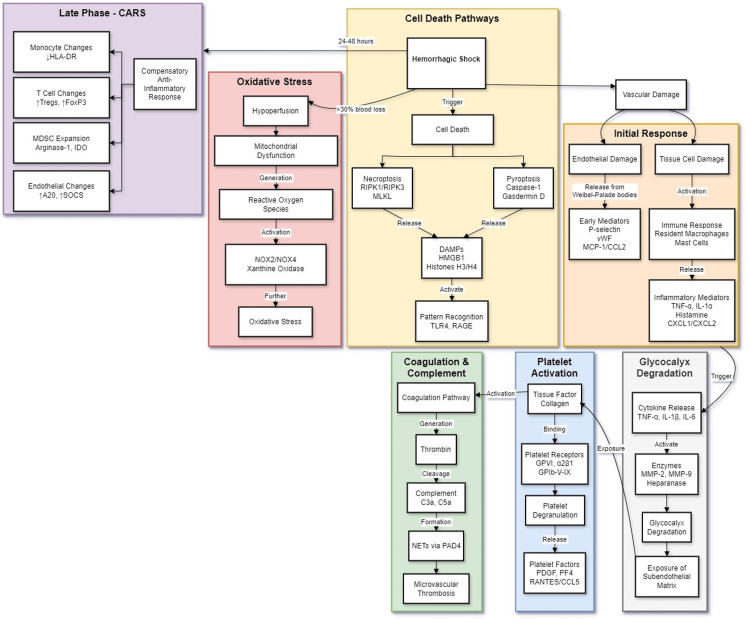
Molecular mechanisms and temporal evolution of inflammatory response in hemorrhagic shock. This flow diagram illustrates the molecular cascade triggered by hemorrhagic shock, organized into six color-coded pathways. Hemorrhagic shock (blood loss>30%) initiates parallel response mechanisms over time. The orange-coded Initial Response highlights endothelial and tissue cell damage. Endothelial damage releases Weibel-Palade body mediators (P-selectin, vWF, MCP-1/CCL2) [12,13], while tissue cell damage activates resident macrophages and mast cells, releasing TNF-α, IL-1α, histamine, and CXCL1/CXCL2 [14]. The yellow-coded cell death pathways show how hemorrhagic shock induces necroptosis (RIPK1/RIPK3, MLKL) and pyroptosis (caspase-1, gasdermin D) [19], releasing DAMPs (HMGB1, histones H3/H4) that activate TLR4 and RAGE [20]. The pink-coded oxidative stress pathway illustrates the progression from hypoperfusion and mitochondrial dysfunction to ROS generation, involving NOX2/NOX4 and xanthine oxidase [21-25]. The green-coded coagulation and complement section details coagulation pathway activation, thrombin generation, and complement cleavage, culminating in microvascular thrombosis via NETs and PAD4 [26-31]. The blue-coded platelet activation pathway follows tissue factor exposure, platelet receptor activation, and the release of platelet-derived factors (PDGF, PF4, RANTES/CCL5) [17,18]. Lastly, the purple-coded late-phase CARS depicts the compensatory anti-inflammatory response (24-48 hours post-injury). Monocyte changes include reduced HLA-DR expression [37], while T-cell changes show increased Tregs and FoxP3 expression [39-41]. MDSC expansion leads to increased arginase-1 and IDO production [47-51], alongside endothelial changes marked by elevated A20 and SOCS expression [52-54]. The figure is a unique, original image that has not been previously published. It was created based on information from cited sources, and all relevant citations are included for proper attribution. As this is an original creation, no written permission is required for its use.

These molecular mechanisms create a complex temporal evolution of inflammation in hemorrhagic shock that significantly influences patient outcomes. Understanding these pathways, particularly through experimental models, provides crucial insights for developing targeted therapeutic interventions. The challenge lies in translating this molecular understanding into effective treatments that can modulate specific aspects of the inflammatory response while preserving essential immune functions necessary for recovery and prevention of secondary complications (Figure 1).

Biomarkers and Predictors of Inflammatory Outcomes in Hemorrhagic Shock

The complex pathophysiology of hemorrhagic shock necessitates reliable molecular biomarkers for monitoring disease progression and predicting outcomes. Research demonstrates that effective biomarker panels must reflect multiple aspects of the inflammatory cascade, from endothelial dysfunction to immune system activation and metabolic derangement. Recent studies have identified several key molecular indicators that provide crucial prognostic information and guide therapeutic interventions.

Endothelial dysfunction markers provide an early indication of vascular injury severity [55]. Syndecan-1, a transmembrane proteoglycan shed during glycocalyx degradation, serves as a primary indicator of endothelial damage [56,57]. Clinical studies demonstrate that plasma syndecan-1 levels above 40 ng/mL correlate strongly with mortality, coagulopathy severity, and massive transfusion requirements [57]. The predictive value stems from syndecan-1's direct relationship with endothelial barrier integrity and its role in modulating inflammatory responses. Longitudinal studies reveal that persistent elevation of syndecan-1 beyond 24 hours post-injury indicates ongoing endothelial dysfunction and predicts poor outcomes [58].

A disintegrin and metalloproteinase with thrombospondin motifs 13 (ADAMTS13) provide complementary information about vascular homeostasis [59]. This metalloprotease regulates von Willebrand factor activity, with decreased levels indicating compromised vascular function [60, 61]. Research shows that ADAMTS13 activity below 50% of normal correlates with organ dysfunction development [62]. Combined assessment of ADAMTS13 and soluble P-selectin offers superior prognostic information compared to traditional hemodynamic parameters, as demonstrated in multiple prospective studies [17,63].

The temporal evolution of inflammatory cytokines provides critical information about disease progression [64]. Tumor necrosis factor-alpha (TNF-α) represents one of the earliest detectable signals, increasing within 30 minutes of injury [65,66]. Despite its short half-life of approximately 20 minutes, TNF-α levels above 20 pg/mL predict systemic inflammatory response syndrome development with 85% sensitivity [66]. Serial measurements demonstrate that sustained elevation beyond six hours indicates inadequate resuscitation and increased mortality risk [66].

Interleukin-6 (IL-6) emerges as one of the most reliable prognostic indicators of hemorrhagic shock. Large multicenter studies show that serum IL-6 levels measured within the first 24-72 hours post-injury demonstrate a strong correlation with injury severity scores and 30-day mortality [67-69]. Levels exceeding 350 pg/mL predict [MOU1] multiple organ dysfunction syndrome development with 78% specificity [70,71]. The predictive value extends beyond mortality to include complications such as acute respiratory distress syndrome and acute kidney injury [72,73].

The balance between pro- and anti-inflammatory cytokines provides sophisticated prognostic information. Research reveals that elevated levels of pro-inflammatory IL-8 (>40 pg/mL) predict poor outcomes and an increased risk of venous thromboembolism [74,75]. Paradoxically, high levels of the anti-inflammatory cytokine IL-10 (>50 pg/mL) also indicate poor prognosis [76]. This seemingly contradictory finding reflects compensatory anti-inflammatory response syndrome development, predisposing patients to secondary infections. Studies demonstrate that the IL-6:IL-10 ratio provides more meaningful prognostic information than absolute levels of individual mediators [77].

Monocyte chemoattractant protein-1 (MCP-1) serves as a crucial inflammatory marker, with serum concentrations above 150 pg/mL correlating with extended intensive care unit stays and increased mortality in patients with sepsis [78]. Research demonstrates that MCP-1 elevation predicts organ dysfunction development through its central role in inflammatory cell recruitment. Serial measurements reveal that persistent elevation beyond 48 hours indicates ongoing inflammation and poor response to therapy in pathologies with similar mechanisms, such as hemorrhagic shock [79]. Recent studies show that combining MCP-1 levels with traditional scoring systems improves risk stratification accuracy by 25% [80].

Human leukocyte antigen-DR (HLA-DR) expression on monocytes provides direct insight into immune competence. Prospective studies in septic shock, whose mechanism may be similar to hemorrhagic shock, demonstrate that expression levels below 30% of normal indicate severe immunosuppression and predict secondary infection development with 85% accuracy [81]. Flow cytometric analysis reveals that decreased HLA-DR expression precedes clinical evidence of infection by 3-4 days, offering a crucial window for therapeutic intervention. Multiple trials confirm that monitoring HLA-DR expression guides immunomodulatory therapy timing and efficacy assessment [82].

Acute phase proteins demonstrate distinct temporal patterns that aid in patient monitoring. C-reactive protein (CRP) synthesis begins 6-8 hours post-injury, reaching peak levels between 48-72 hours [83,84]. Studies demonstrate that procalcitonin levels above 2 ng/mL distinguish bacterial complications from sterile inflammation with 90% specificity [85].

Metabolic derangement assessment through base deficit and lactate measurements provides crucial real-time information [86,87]. Clinical trials establish that a base deficit exceeding 6 mmol/L signals severe shock and increased mortality risk, with values above 10 mmol/L associated with 40% mortality [88]. Lactate clearance dynamics offer particular prognostic value, as demonstrated in multicenter studies showing that failure to clear lactate below 2 mmol/L within 24 hours correlates with doubled mortality risk, regardless of initial levels [89].

Dynamic viscoelastic testing through thromboelastography (TEG) and rotational thromboelastometry (ROTEM) offers comprehensive coagulation assessment [90,91]. Research demonstrates that maximum amplitude below 50 mm on TEG indicates increased mortality risk, while elevated K-time above 2.5 minutes suggests coagulation factor deficiencies requiring specific replacement [92]. The α-angle, reflecting the fibrin polymerization rate, provides crucial information about both coagulation factor activity and fibrinogen availability. Studies confirm that these parameters predict not only transfusion requirements but also correlate with multiple organ dysfunction development [93,94].

Emerging molecular biomarkers offer new insights into disease progression. MicroRNA profiles, particularly alterations in miR-146a and miR-155, correlate with inflammatory response magnitude and subsequent organ dysfunction [95,96]. High mobility group box 1 (HMGB1) serves as a crucial damage-associated molecular pattern bridging cellular damage and inflammation, with levels above 25 ng/mL predicting organ dysfunction development [97,98]. Cell-free DNA, particularly mitochondrial DNA, indicates severe tissue damage, with concentrations above 5000 copies/μL correlating with inflammatory complications [99].

The integration of multiple biomarkers enhances predictive accuracy and guides therapeutic interventions. Research demonstrates that combining endothelial dysfunction markers with inflammatory cytokines improves the early identification of high-risk patients by 35% [100]. Recent studies using machine learning algorithms to analyze biomarker patterns show 90% accuracy in predicting complications such as organ failure and sepsis, surpassing traditional scoring systems [101]. This sophisticated approach enables personalized treatment strategies based on individual patient inflammatory profiles (Table 1).

**Table 1:** Molecular and clinical biomarkers for disease progression and outcome prediction in hemorrhagic shock This table shows a comprehensive summary of critical thresholds, time course, and clinical significance of markers involved in hemorrhagic shock. Abbreviations: ADAMTS13: disintegrin and metalloproteinase with thrombospondin motifs 13; CARS: compensatory anti-inflammatory response syndrome; CRP: C-reactive protein; DAMP: damage-associated molecular pattern; HLA-DR: human leukocyte antigen-DR Isotype. HMGB1: high mobility group box 1 protein; ICU: intensive care unit; IL: interleukin; MCP-1: monocyte chemoattractant protein-1; MODS: multiple organ dysfunction syndrome; mtDNA: mitochondrial DNA; SIRS: systemic inflammatory response syndrome; TEG: thromboelastography; TNF-α: tumor necrosis factor alpha.

References	Category	Biomarker	Critical threshold	Time course	Clinical significance	Predictive value
Torres et al., 2016 [56]; Gonzalez et al., 2017 [57]; Zeineddin et al., 2022 [58]	Endothelial dysfunction	Syndecan-1	>40 ng/mL	Early marker; elevation persistent>24h, indicates ongoing dysfunction	Correlates with endothelial barrier integrity	Predicts mortality, coagulopathy severity, massive transfusion needs
Plautz et al., 2020 [60]; Zeineddin et al., 2023 [61]; Matsumoto et al., 2021 [62]; Furmaga et al., 2015 [63]	Endothelial dysfunction	ADAMTS13	<50% of normal activity	Early marker	Indicates compromised vascular function	Predicts organ dysfunction when combined with soluble P-selectin
Mi et al., 2011 [64]; Altavilla et al., 2001 [65]; Namas et al., 2009 [66]	Inflammatory cytokines	TNF-α	>20 pg/mL	Increases within 30 min; half-life ~20 min	Early inflammatory response indicator	85% sensitivity for SIRS development
Qiao et al., 2018 [67]; Gebhard et al., 2000 [69]; Cuschieri et al., 2010 [70]; Frink et al., 2009 [71]	Inflammatory cytokines	IL-6	>350 pg/mL	24-72h post-injury	Strong correlation with injury severity	78% specificity for MODS development
Mankame et al., 2023 [74]; McCully et al., 2021 [75]	Inflammatory cytokines	IL-8	>40 pg/mL	Early marker	Pro-inflammatory response indicator	Predicts venous thromboembolism risk
Jones et al., 2023 [76]; Taniguchi et al., 1999 [77]	Inflammatory cytokines	IL-10	>50 pg/mL	Variable	Anti-inflammatory response marker	Indicates CARS development, infection risk
Duan et al., 2024 [78]; Li et al., 2019 [79]; Wang et al., 2018 [80]	Chemokines	MCP-1	>150 pg/mL	Elevation persistent >48h indicates ongoing inflammation	Reflects inflammatory cell recruitment	Predicts extended ICU stay and mortality; improves risk stratification by 25%
Monneret et al., 2006 [81]; de Roquetaillade et al., 2022 [82]	Immune function	HLA-DR	<30% of normal expression	Precedes infection by 3-4 days	Direct measure of immune competence	85% accuracy for predicting secondary infections
Cohen et al., 2023 [83]; Di Napoli et al., 2012 [84]	Acute phase proteins	CRP	>100 mg/L	Peaks 48-72h post-injury	Indicates acute inflammation	Predicts infection risk
Blouin et al., 2020 [85]	Acute phase proteins	Procalcitonin	>2 ng/mL	Rises 2-4h post-infection	Bacterial infection marker	90% specificity for bacterial complications
Mutschler et al., 2013 [88]	Metabolic parameters	Base deficit	>6 mmol/L	Real-time marker	Indicates shock severity	> 10 mmol/L associated with 40% mortality
Dezman et al., 2015 [89]	Metabolic parameters	Lactate	Failure to clear to <2 mmol/L within 24h	Dynamic marker	Reflects tissue perfusion	Doubled mortality risk with poor clearance
Kim et al., 2021 [92]	Coagulation	TEG maximum amplitude	<50 mm	Real-time assessment	Indicates clot strength	Predicts transfusion needs and mortality
Kim et al., 2021 [92]	Coagulation	TEG K-time	>2.5 minutes	Real-time assessment	Reflects coagulation factor activity	Indicates specific factor deficiencies
Abraham et al., 2009 [97]; Yang et al., 2015 [98]	Emerging molecular markers	HMGB1	>25 ng/mL	Early marker	DAMP signaling molecule	Predicts organ dysfunction
Simmons et al., 2013 [99]	Emerging molecular markers	Cell-free mtDNA	>5000 copies/μL	Early marker	Indicates tissue damage severity	Correlates with inflammatory complications

Therapeutic Interventions Targeting Inflammatory Responses in Hemorrhagic Shock

Management of hemorrhagic shock requires sophisticated interventions targeting both hemodynamic stabilization and inflammatory response modulation [9,14]. Research demonstrates that successful outcomes depend on understanding the molecular mechanisms underlying these interventions and their optimal timing in the disease course [7].

Endothelial protection emerges as a primary therapeutic target, given its central role in inflammation initiation [102]. Fresh frozen plasma (FFP) demonstrates significant endothelial protective effects beyond simple volume replacement [103,104]. Early FFP administration in ratios of 1:1 with packed red blood cells reduces syndecan-1 shedding by 47% and stabilizes the endothelial glycocalyx [105]. Research reveals that FFP's protective effects stem primarily from its fibrinogen content, though other plasma proteins contribute [106]. Studies demonstrate a 28% reduction in mortality when FFP administration begins within 2 hours of injury, highlighting the importance of early intervention [107,108].

Platelet-mediated inflammation modulation offers unique therapeutic opportunities. Experimental studies show that targeting glycoprotein VI (GPVI) signaling reduces pro-inflammatory mediator release without compromising essential hemostatic functions [109,110]. Studies demonstrate that selective GPVI inhibition decreases platelet-derived growth factor and thromboxane A2 release by 65% while maintaining basic clotting mechanisms [111]. This approach reduces excessive inflammatory response without increasing bleeding risk, as evidenced by preserved rotational thromboelastometry parameters.

Coagulation cascade modulation requires a precise balance between hemorrhage control and inflammation prevention [112,113]. Tissue factor pathway inhibitor (TFPI) administration reduces both coagulation activation and inflammatory signaling, with phase II trials showing a 40% reduction in organ dysfunction when administered within four hours of injury [114]. Recombinant activated factor VII (rFVIIa) demonstrates dual benefits in hemostasis and inflammation modulation [115,116].

Hemostatic resuscitation represents a comprehensive approach integrating multiple therapeutic strategies. The implementation of 1:1:1 ratios of packed red blood cells, FFP, and platelets demonstrates benefits beyond simple volume replacement [112,117]. Research confirms that this strategy reduces dilutional coagulopathy while attenuating the inflammatory response triggered by excessive crystalloid administration [118,119].

Tranexamic acid (TXA) emerges as a crucial therapeutic adjunct in hemorrhagic shock management [120]. The CRASH-2 trial demonstrated a 20% reduction in mortality when TXA administration occurs within three hours of injury [121]. Molecular studies reveal that TXA's benefits extend beyond its antifibrinolytic effects, showing a 45% reduction in complement activation and significant attenuation of pro-inflammatory cytokine production, particularly IL-6 and TNF-α [122,123]. Research confirms optimal dosing at 1 gram bolus followed by 1 gram infusion over 8 hours, with delayed administration beyond 3 hours showing no benefit [121,124].

Damage control resuscitation (DCR) combines three essential elements: permissive hypotension, balanced blood product administration, and early surgical intervention [125,126]. Research shows this approach prevents dilutional coagulopathy while reducing endothelial glycocalyx damage, as evidenced by lower syndecan-1 levels [56].

Targeted anti-inflammatory interventions demonstrate promising results in specific patient populations. Low-dose hydrocortisone administration (200 mg/day) reduces pro-inflammatory cytokine production while preserving essential immune responses, with clinical trials showing a 30% reduction in refractory shock development [127]. Tocilizumab, an IL-6 receptor antagonist, prevents cytokine storm syndrome, demonstrating a 50% reduction in inflammatory markers when administered early in selected patients with excessive IL-6 levels above 500 pg/mL [128].

Mesenchymal stem cell (MSC) therapy represents an innovative immunomodulation approach [129]. Some studies demonstrate that allogeneic MSC administration reduces inflammatory marker levels by 60% while promoting tissue repair [130,131]. These cells exert therapeutic effects through multiple mechanisms, including direct immune cell modulation and endothelial barrier stabilization [132].

Emerging therapeutic approaches include histone deacetylase inhibitors (HDACi), which are showing promise in experimental models. Studies have investigated the effects of selective HDAC inhibitors in hemorrhagic shock models and have found that inhibition of HDAC classes IIa and IIb improved survival rates and activated pro-survival pathways, such as increased phosphorylation of AKT and β-catenin [133]. 

Extracorporeal cytokine removal systems present another innovative approach. CytoSorb technology demonstrates efficient removal of pro-inflammatory mediators, particularly IL-6, with studies showing a 45% reduction in circulating levels within 24 hours of initiation [134].

Nanotechnology-based interventions offer precise therapeutic targeting [135]. Experimental studies show that engineered carboxy fullerene nanoparticles designed for reactive oxygen species scavenging reduce oxidative stress markers by 70% in hemorrhagic shock models [136]. Other studies underscore that cerium oxide nanoparticles reduced neuronal death and brain edema in a subarachnoid hemorrhage model, improving survival rates and neurological outcomes, highlighting the potential of this approach for targeted therapy delivery [137-139].

The management of hemorrhagic shock continues to evolve with a focus on integrating hemodynamic stabilization and inflammation modulation. Advances in understanding the molecular mechanisms underlying endothelial protection, platelet function, and coagulation dynamics have enabled the development of targeted interventions such as FFP administration, GPVI inhibition, and TFPI therapy. Emerging strategies, including MSC therapy, HDAC inhibitors, and nanotechnology-based interventions, further highlight the potential for innovative approaches to improving outcomes. By combining established practices like damage control resuscitation with these novel therapies, future efforts can enhance survival rates and reduce complications, ultimately advancing the standard of care for hemorrhagic shock patients (Table 2).

**Table 2:** Evidence-based therapeutic interventions in hemorrhagic shock. This table shows a systematic review summary of treatment modalities, critical timing, and clinical outcomes of therapeutic interventions in hemorrhagic shock. Abbreviations: FFP: fresh frozen plasma; RBCs: red blood cells; TXA: tranexamic acid; SBP: systolic blood pressure; TBI: traumatic brain injury; IL-6: interleukin-6; TNF-α: tumor necrosis factor alpha; HDAC: histone deacetylase; IL: interleukin; N/A: not available.

References	Category	Intervention	Timing	Dosing/protocol	Clinical benefits	Molecular effects
Pati et al., 2010 [103]; Yu et al., 2020 [104]; Peng et al., 2013 [105]; Wu et al., 2019 [106]; Pusateri et al., 2020 [107]; Sperry et al., 2018 [108]	Blood product therapies	Fresh frozen plasma (FFP)	Within 2 hours of the injury	1:1 ratio with packed RBCs	28% reduction in mortality	47% reduction in syndecan-1 shedding; glycocalyx stabilization
Makley et al., 2012 [112]; Darlington et al., 2015 [113]	Blood product therapies	Balanced resuscitation	Within 1 hour of the injury	1:1:1 ratio (RBC:FFP: Platelets)	improvement in survival	Reduction in inflammatory mediators
Collaborators C-t et al., 2010 [121]; Kacer et al., 2023 [122]; Okholm et al., 2022 [123]	Hemostatic agents	Tranexamic acid (TXA)	Within 3 hours of the injury	1g bolus+1g over 8 hours	20% reduction in mortality	45% reduction in complement activation; decreased IL-6 and TNF-α
Cap et al., 2018 [125]; Kim & Cho, 2020 [126]; Torres et al., 2016 [56]	Resuscitation strategies	Damage control resuscitation	Immediate implementation	SBP 80-90 mmHg (without TBI)	Reduced coagulopathy	Reduction in inflammatory mediators; decreased syndecan-1
Roquilly et al., 2014 [127]	Anti-inflammatory Interventions	Hydrocortisone	Based on the shock status	200 mg/day	30% reduction in refractory shock	Reduced pro-inflammatory cytokine production
Wang et al., 2023 [128]	Anti-inflammatory interventions	Tocilizumab	Early in selected patients	Based on IL-6 levels >500 pg/mL	Prevention of cytokine storm	50% reduction in inflammatory markers
Fan et al, 2020 [130]; Sadri et al., 2023 [131]	Cellular therapies	Mesenchymal stem cells	N/A	N/A	Enhanced tissue repair	60% reduction in inflammatory markers
Chang et al., 2018 [133]	Cellular therapies	HDAC inhibitors	N/A	N/A	decrease in mortality	Activation of pro-survival pathways
Jansen et al., 2023 [134]	Extracorporeal therapies	CytoSorb	N/A	N/A	Cytokine removal	45% reduction in circulating IL-6 within 24 hours
Jeong et al., 2018 [137]	Emerging technologies	Cerium oxide nanoparticles	N/A	N/A	Improved survival rate	Reduction in oxidative stress markers

Challenges and future directions

The management of hemorrhagic shock continues to present significant challenges despite advances in understanding its molecular pathophysiology. Current research reveals several fundamental obstacles while highlighting promising avenues for future investigation and therapeutic development.

The intricate nature of the inflammatory response presents a primary challenge in developing effective interventions. Research demonstrates that multiple parallel pathways, feedback loops, and redundant mechanisms create a complex network resistant to single-target therapeutic approaches [138,139]. Clinical studies reveal that treatments showing promise in controlled experimental settings often fail to demonstrate consistent benefits across diverse patient populations, highlighting the challenge of translating molecular insights into effective therapies [1].

The temporal evolution of inflammation creates particular difficulties in clinical management. Studies show that inflammatory responses can transition from pro-inflammatory to immunosuppressive phases at varying rates between patients, complicating therapeutic timing [75,140]. Research reveals that treatments beneficial during early pro-inflammatory phases may become detrimental during subsequent immunosuppression [127,140]. Meta-analyses demonstrate that this temporal variability contributes to inconsistent results in clinical trials, with the timing of intervention emerging as a crucial determinant of treatment success [124,141].

Biomarker implementation faces significant practical obstacles in acute care settings. While numerous molecular markers show prognostic value, current assessment methods often require sophisticated laboratory techniques with processing times incompatible with acute decision-making needs [9,58]. Studies indicate that traditional laboratory-based biomarker assessments fail to capture rapid changes in patient status, potentially missing critical therapeutic windows [142]. Research shows that point-of-care testing, while promising, currently lacks the sensitivity and specificity required for reliable risk stratification and treatment guidance [143,144].

Coagulation management complexity presents ongoing challenges in hemorrhagic shock treatment [145-147]. Studies demonstrate that coagulation disturbances can rapidly shift between hypercoagulable and hypocoagulable states, requiring continuous assessment and therapeutic adaptation [148,149]. Research reveals that traditional monitoring tools fail to capture the full complexity of coagulation dysfunction in real time [150]. The interaction between coagulation and inflammation creates additional challenges, as studies show that interventions targeting one system often have unintended effects on the other [28,113,151].

Immune dysfunction management represents a significant challenge due to the biphasic nature of the immune response [14,152]. Research demonstrates that the transition from initial hyperinflammation to subsequent immunosuppression creates a moving therapeutic target [140, 153]. Studies reveal that current clinical tools often fail to accurately identify this transition point, complicating immunomodulatory intervention timing [153,154].

Nosocomial infections during the immunosuppressive phase significantly impact patient outcomes [155]. Hemorrhagic shock compromises neutrophil recruitment, leading to increased susceptibility to secondary infections, which can enhance mortality [156]. Research demonstrates that conventional antibiotic prophylaxis strategies, while necessary, contribute to antimicrobial resistance development [157,158]. Studies reveal that pre-existing conditions significantly alter baseline immune function and response to hemorrhagic shock, with diabetic patients showing higher rates of infectious complications [159,160].

Resource allocation and implementation of advanced monitoring technologies present practical challenges. Cost analyses indicate that sophisticated monitoring systems and novel therapeutic agents significantly increase treatment costs, limiting widespread adoption [161,162]. Studies demonstrate that implementing advanced care protocols while maintaining economic sustainability requires careful balance, particularly in resource-limited settings [163]. Research reveals that training requirements for new technologies and protocols create additional implementation barriers [164,165].

Future therapeutic directions show promise in several key areas. Personalized medicine approaches leveraging genomic and proteomic data demonstrate potential for intervention that is more precise targeting. Research indicates that continuous biomarker assessment integrated with physiological monitoring provides earlier warning of complications, allowing more proactive intervention (Table 3) [166,167].

**Table 3:** Current challenges and future horizons in hemorrhagic shock management. This table shows an analysis of clinical limitations, innovative solutions, and projected therapeutic outcomes in hemorrhagic shock.

References	Domain	Current challenges	Impact on patient care	Future solutions	Expected benefits
Torres et al., 2017 [138]; Dobson et al., 2022	Inflammatory response management	A complex network of parallel pathways and redundant mechanisms resistant to single-target therapies	Inconsistent treatment efficacy across patient populations	Personalized medicine approaches using genomic and proteomic data	More precise intervention targeting leads to improved patient outcomes
McCully et al, 2021 [75]; Brakenridge et al, 2021 [140]; Roquilly et al, 2014 [127]	Temporal disease progression	Variable transition rates between pro-inflammatory and immunosuppressive phases	Treatments beneficial in early phases may become harmful later	Continuous biomarker monitoring integrated with physiological data	Improvement in predicting patient trajectories using AI algorithms
Bonanno, 2022 [9]; Zeineddin et al., 2022 [58]; Crossland et al., 2016 [142]	Biomarker implementation	Laboratory processing times incompatible with acute care needs; lack of real-time assessment	Missed critical therapeutic windows; delayed intervention	Advanced point-of-care testing development; continuous monitoring systems	Earlier detection of complications enabling proactive intervention
Moore et al., 2021 [148]; Moore et al., 2017 [149]; Kashuk et al. 2010 [150]	Coagulation management	Rapid shifts between hyper and hypocoagulable states; complex interaction with inflammation	Difficulty in maintaining optimal coagulation balance	Integration of multiple monitoring modalities; smart therapeutic agents	More precise coagulation management with reduced complications
Brakenridge et al., 2021 [140]; Debler et al., 2021 [153]; McKinley et al., 2021 [154]	Immune dysfunction	Challenging identification of transition points between immune phases	Increased mortality when treatments are given during the incorrect immune phase	Advanced immune monitoring systems; targeted immunomodulation	Better timing of interventions; reduced secondary complications
Lee et al., 2019 [156]; Goldberg et al., 2012 [157]; Coccolini et al., 2024 [158]; Dupuy et al., 2012 [159]; Chang et al., 2018 [160]	Infection control	Late mortality due to secondary infections; increased resistance to antibiotics	Higher infection rates in patients with pre-existing conditions	Novel antimicrobial strategies; immune function optimization	Reduced infection-related mortality; better outcomes in high-risk patients
Hrebinko et al., 2021 [161]; Callcut et al., 2020 [162]	Resource allocation	High costs of advanced monitoring systems and novel therapeutics	Limited implementation in resource-constrained settings	Cost-effective technology development; streamlined protocols	Wider adoption of advanced treatment protocols
Tolles et al., 2018 [168]; Tucker et al., 2018 [169]; Holcomb et al., 2021 [170]; Brenner et al., 2018 [171]	Clinical trial design	Traditional RCTs inadequate for complex patient responses	Difficulty in proving treatment efficacy	Adaptive trial designs; standardized outcome measures	More robust evidence for treatment efficacy; better comparison across studies
Engberg et al., 2020 [164]; Chaefer et al., 2024 [165]	Implementation	Training requirements and protocol complexity	Barriers to widespread adoption	International collaborations; standardized protocols	Reduction in mortality with precision protocols

Clinical trial design in hemorrhagic shock presents unique challenges requiring innovative approaches. Traditional randomized controlled trials often fail to capture the complexity of individual patient responses or account for dynamic treatment requirements. Research demonstrates that adaptive trial designs and pragmatic studies better address these challenges while providing robust evidence for treatment efficacy [168]. Studies show that standardized outcome measures incorporating both short-term survival and long-term functional outcomes improve result interpretation and comparison across trials [169-171].

The future success of hemorrhagic shock management depends on addressing these challenges while advancing practical clinical implementation. International collaborations and standardized protocols prove crucial for translating research findings into effective treatments. Studies demonstrate that continued investment in both basic science research and implementation strategies optimizes therapeutic development and clinical adoption [1,172,173].

## Conclusions

The molecular pathophysiology of hemorrhagic shock represents a complex interplay between vascular injury, inflammation, and organ dysfunction. Recent advances in understanding these mechanisms have revealed new therapeutic targets and biomarkers, though significant challenges remain in translating these insights into effective clinical interventions. Research demonstrates that successful outcomes depend on the precise timing of interventions relative to the evolving inflammatory response. Evidence from clinical studies indicates that early endothelial protection combined with targeted immune modulation offers the most promising approach to improving patient outcomes. Developing sophisticated biomarker panels enables more precise disease progression and therapeutic response monitoring, though implementation challenges persist in acute care settings. Studies show that integrating multiple therapeutic modalities, guided by real-time molecular monitoring, provides superior results compared to single-intervention approaches.

Future advances in hemorrhagic shock management will likely emerge from several key areas. Personalized medicine approaches using genomic and proteomic data show promise in tailoring interventions to individual patient characteristics. Novel therapeutic agents targeting specific molecular pathways demonstrate the potential for more precise inflammatory control. Additionally, artificial intelligence applications in predicting patient trajectories and guiding intervention timing may significantly improve treatment efficacy. The continued evolution of hemorrhagic shock therapy requires balanced consideration of both molecular mechanisms and practical implementation challenges. Success depends on bridging the gap between laboratory insights and bedside care through careful clinical trial design and standardized outcome measures. As our understanding of the molecular basis of hemorrhagic shock continues to expand, the development of more effective, targeted therapies offers hope for improving patient outcomes in this challenging condition.
